# Evaluating Reticulorumen Temperature, Rumination, Activity and pH Measured by Rumen Sensors as Indicators of Heat Load in Fattening Bulls

**DOI:** 10.3390/s25206401

**Published:** 2025-10-16

**Authors:** Kay Fromm, Christian Ammon, Thomas Amon, Gundula Hoffmann

**Affiliations:** 1 Department of Sensors and Modelling, Leibniz Institute for Agricultural Engineering and Bioeconomy e.V., Max-Eyth-Allee 100, 14469 Potsdam, Germany; cammon@atb-potsdam.de (C.A.); tamon@atb-potsdam.de (T.A.); ghoffmann@atb-potsdam.de (G.H.); 2 Department of Veterinary Medicine, Institute of Animal Hygiene and Environmental Health, Freie Universität Berlin, Robert-von-Ostertag-Str. 7-13, 14163 Berlin, Germany

**Keywords:** rumen sensor-based monitoring, precision livestock farming, bull husbandry, heat load detection, reticulorumen temperature, rumen pH

## Abstract

The aim of this experiment was to determine whether reticulorumen temperature (ReT), rumination, activity or pH captured by a rumen sensor bolus system (smaXtec animal care GmbH, Graz, Austria) can be used as an early indicator of heat load (HL) and to assess how its daily patterns are influenced by diurnal effects. Physiological and behavioral data from 70 male feedlot cattle (Uckermärker, Hereford, Simmentaler) housed in a closed barn were investigated using the calculated temperature-humidity index (THI) from remote HOBO Onset climate sensors over a period of 210 days. Using time series analysis and seasonal ARIMA modeling, it was found that ReT followed the same patterns throughout days with a THI < 74 as well as days under heat load conditions. Time series and correlation analyses were also performed for the rumen pH, rumination index and activity index. The collective mean ReT over the winter days assessed (*n* = 14,971) was 39.48 °C, with a minimum mean of 38.31 °C and a maximum mean of 40.69 °C. In comparison, the collective mean ReT over the summer days assessed (*n* = 14,030) was 39.53 °C, with a minimum mean of 38.39 °C and a maximum mean of 42.02 °C. Pearson’s correlation did not reveal a relationship between THI and ReT (*r* = −0.06; *p* < 0.001) and only minimally for rumination (*r* = −0.11; *p* < 0.001). Rumination clearly decreased with increasing ambient temperature in comparison to days with a THI < 74. A long-term effect is also visible when the monthly mean rumination from all bulls tends to decrease slightly from February to May and then increases beginning in June. The mean pH values decreased throughout the summer months. Nevertheless, the comparison between daily fluctuations in pH values under HL failed to yield significant deviations from those captured on days of winter. The Pearson correlation for rumen pH showed a weak negative linear relationship with THI (*r* = −0.3; *p* < 0.001). The monthly means of the motion activity index could also not verify that HL led to increasing activity (Pearson correlation for motion activity and THI: *r* = 0.04; *p* < 0.001). The heat load had no visible short-term effects on the ReT or rumen pH, but rumination and peak motion activity were reduced on days with high ambient temperatures.

## 1. Introduction

Thermal changes in the global climate influence animal husbandry systems from an economic perspective because they can negatively impact health and welfare conditions. In the past two to three decades, the climate has undergone major changes and become more unpredictable, significantly affecting agri-animal husbandry. Several international agencies, such as the Intergovernmental Panel on Climate Change (IPCC) and the United States Environmental Protection Agency (USEPA), have predicted a steady increase in ambient temperatures [1,2]. Possible consequences are decreased food intake and resting periods, which cause a loss in productivity. Previous studies estimated annual losses due to heat stress (HS) of US$1.26 billion for dairy and beef cattle herds in the USA in the early 2000s and income losses of £40 million in the UK dairy herd in some years by 2080 in the absence of mitigation measures [3,4,5,6]. Studies have demonstrated that HS has a negative effect on meat color and water-holding capacity and results in higher postmortem ultimate pH values in cattle and small ruminants [7,8,9,10,11]. Most studies on the effects of HS have focused on production inefficiencies such as reduced milk yield and death in mature dairy and beef cattle [12,13,14,15,16,17]. However, scientific evidence has shown that HS influences the physiology, feed conversion efficiency, rumen and reproduction of calves and heifers [18]. Regarding investigations on the physiological responses to HS in bulls and steers, the focus is mainly on semen quality and reproductive health [19,20,21,22,23,24,25]. High-producing animals are already at the threshold of their maximum production and generate more heat energy, which continues to accumulate if the environmental conditions are adverse [26]. Therefore, the more an animal produces, the more metabolic heat it generates, and additional thermoregulatory pathways are triggered to maintain homoeothermic conditions, increasing the vulnerability of the animal to HL [27]. To minimize these scenarios, it is highly important for scientists, stakeholders and farmers to develop methods to detect HL at an early stage before it affects animals.

Markers for HL can include the respiration rate, lying duration, rumination time, and drinking cycle. Of course, the outside temperature can also be used as an indicator of HL. In combination with the humidity, the temperature-humidity index (THI) can be calculated and predicted. Species-dependent THI values strongly suggest the development of HL and should be taken as a late warning factor. Only physiological parameters can provide individual animal HS predictors.

The temperature of the animals can be seen as one of the controversial physiological parameters used for HL detection. Although an increase in core temperature would suggest the appearance of HL, studies could not completely confirm this hypothesis [28]. One problem was the lack of data from measuring core temperature continuously in beef cattle without disturbing the animal. An aim of the study was to close this gap with a rumen sensor bolus.

The aim of our study was to investigate whether we can utilize this sensor system to detect HL in fattening bulls by observing the reticulorumen temperature (ReT) of the animals under mild conditions (THI < 74) and conditions that definitely resulted in heat load (THI ≥ 74). Additionally, we traced rumination, activity and pH and investigated their possible associations with HL. Furthermore, whether the individual animal data were sufficiently sensitive to detect HL in an early state or could only provide evidence that bulls experienced HL in retrospect was highly interesting.

## 2. Materials and Methods

### 2.1. Animals and Housing

**Figure 1:**
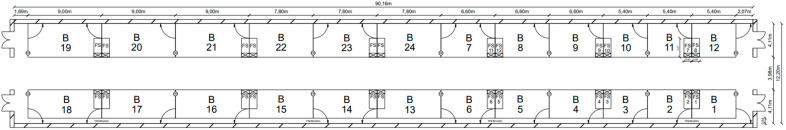
Floor plan of the barn for feedlot cattle; B = box/pen, FS = feeding station.

This study was conducted at the Educational and Experimental Center for Animal Breeding and Husbandry (LVAT; Groß Kreutz, Germany) from December 2022 to July 2023. In total, 70 bulls (Uckermärker, Hereford, Simmentaler) were used in this study, and the data were recorded continuously for 210 days. The fattening bulls were housed in a closed barn in pens on a slatted floor with rubber mats in groups of ten bulls with access to two drinking bowls in a space of 49.73 m^2^ (Figure 1). The stabling was staggered, with its beginning in September 2022 until March 2023. At this time, the bulls weighed 300 to 350 kg. At the end of the fattening period (17 to 18 months), they weighed 700 to 800 kg. During the trial, the animals were able to move freely in the pens without disturbance. The bulls were fed ad libitum with TMR (90% mays silage, 7.1% rape extraction meal, 2% straw, 0.6% vilomin, 0.2% feeding lime and 0.1% animal feed salt). The daily provision of feed was provided at 8 a.m. and moved into position at approximately 1 p.m. The parti-cipants also had access to the concentrate feed machine, which was limited to three meals (pellets consisting of living yeast, selenium, other minerals, trace elements and vitamins) a day and 2 kg per bull and meal.

### 2.2. Sensor Data

**Figure 2:**
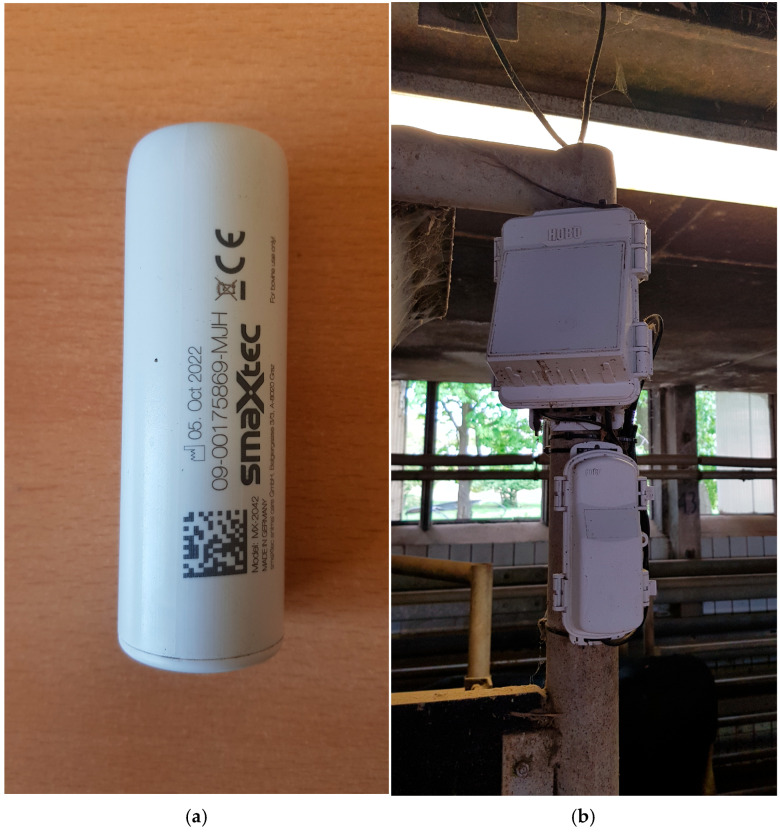
(**a**) Rumen sensor bolus. (**b**) Base station for Hobo Onset climate sensors (air temperature, relative humidity, solar radiation, and wind speed).

The animals were equipped with rumen sensor boluses (smaXtec animal care GmbH, Graz, Austria), which were 210 g in weight and 105 × 35 mm in size. At the age of nine months, the rumen sensor boluses were inserted with a dispenser (by a veterinarian, as suggested by the developers) and placed at the reticulum when the minimum body weight was 300 kg. The non-invasive measures performed on the animals associated with the experimental project were approved by the State Office for Occupational Safety, Consumer Protection, and Health in Brandenburg under the designation V6-2347/0-2022-10. All the data were captured every 10 min and transferred via Wi-Fi to a messenger service (smaXtec messenger^®^ v4.8.21 software, Graz, Austria). The following characteristics were used in the study: reticulorumen temperature in °C, motion activity index (range 0 to 100), and rumination index (lapse of reticular contractions within 24 h in seconds measured by roll-over of the accelerometer). Every reticulorumen temperature drop of at least 1.5 °C from the previously recorded value within a period of no more than 10 min was detected as one drinking cycle (a drinking event lasts on average 1–3 min, and the values are recorded every 10 min) and set aside from the data table. Specifically, the rumen pH was measured in 14 bulls (two per group) using a different bolus from the same developer (length: 120 mm; width: 35 mm; weight: 210 g). pH probes calibration was performed using pH 7 buffer solutions at the beginning of the experiment. Because of its dimensions, this indwelling system can be orally administered to adult cattle and is shockproof and resistant to rumen fluid (Figure 2a).

The temperature and humidity were recorded using a temperature and humidity recorder (Hobo Onset Computer Corporation, 470 MacArthur Blvd, Bourne, MA 02532 USA) continuously from December 2022 until July 2023 every 5 min (Figure 2b). The temperature-humidity loggers were placed above the pens of the tested animals, but out of reach for the bulls, at four points in the barn. The locations of the sensors were equally spaced 22 m from each other. From these four sensors, the median values of temperature and humidity were used to calculate THI. The temperature and humidity index (THI) was calculated using the following formula:

THI = (1.8 × T + 32) − [(0.55 − 0.0055 × RH) × (1.8T − 26)](1)

where T is the temperature (°C) and RH is the relative humidity expressed as a percentage (%) [29,30]. The average daily THI values during the chosen experimental period under summer conditions were greater than 74. THI values were interpreted as follows: ≤74, comfort; 75–78, alert; 79–83, dangerous; and ≥84, emergency [29,31,32].

### 2.3. Statistical Analysis

Statistical analysis was performed using JMP (16.1.0; SAS Institute, Cary, NC, USA) and SAS (9.4; SAS Institute, Cary, NC, USA). For the time series and model of the seasonal ARIMA, only 49 individuals were chosen from the pool because not all the animals were available for winter and summer conditions. For each season, two days with THI values within the comfort (winter; THI < 74) and alert (summer; THI 75–78) situations were selected. With all 70 bulls, a linear model was created to describe and predict the behavior of the complex systems and to analyze the experimental data as follows:

Y_jkl_ = μ + D_j_ + H_k_ + A_l_ + DH_jk_ + HA_kl_ + DA_jl_ + DHA_jkl_ + e_jkl_ (2)

where Y_jkl_ is the dependent variable (reticulorumen temperature, pH, activity, and rumination for the l bull in the j month and the k time point); μ is the general mean; and D_j_ is the day of month (fixed effect, 337 classes); H_k_ is the time (fixed effect, 144 classes); A_l_ is the animal (random effect, 70 classes); DH_jk_ is the interaction D × H; HA_kl_ is the interaction H × A; DA_jl_ is the interaction D × A; DHA_jkl_ is the interaction D × H × A; and e_jkl_ is the residual error.

Pearson’s correlation was performed, given that *x* = THI and *y* = [ReT, rumen pH, rumination index, activity index] with all 70 bulls over the complete time period of 210 days. Further, Pearson’s and Spearman’s correlation was performed, given that *x* = maximum daily THI and *y* = [ReT range, rumen pH range, rumination index range, activity index range] with each individual bull differentiated according to summer and winter conditions.

## 3. Results

### 3.1. Reticulorumen Temperature

**Figure 3:**
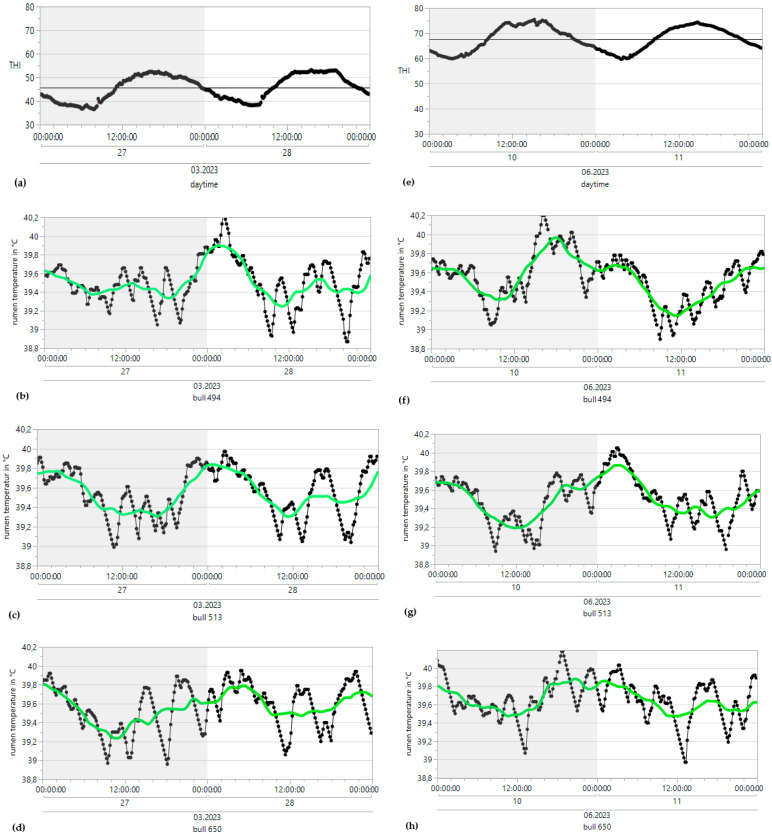
Time series analysis of THI (temperature-humidity index) and rumen temperature (without drinking) under heat stress (**right**) and winter conditions (**left**) from single bulls; simple moving average [SMA (37, centered)] = green curve.

**Figure 4:**
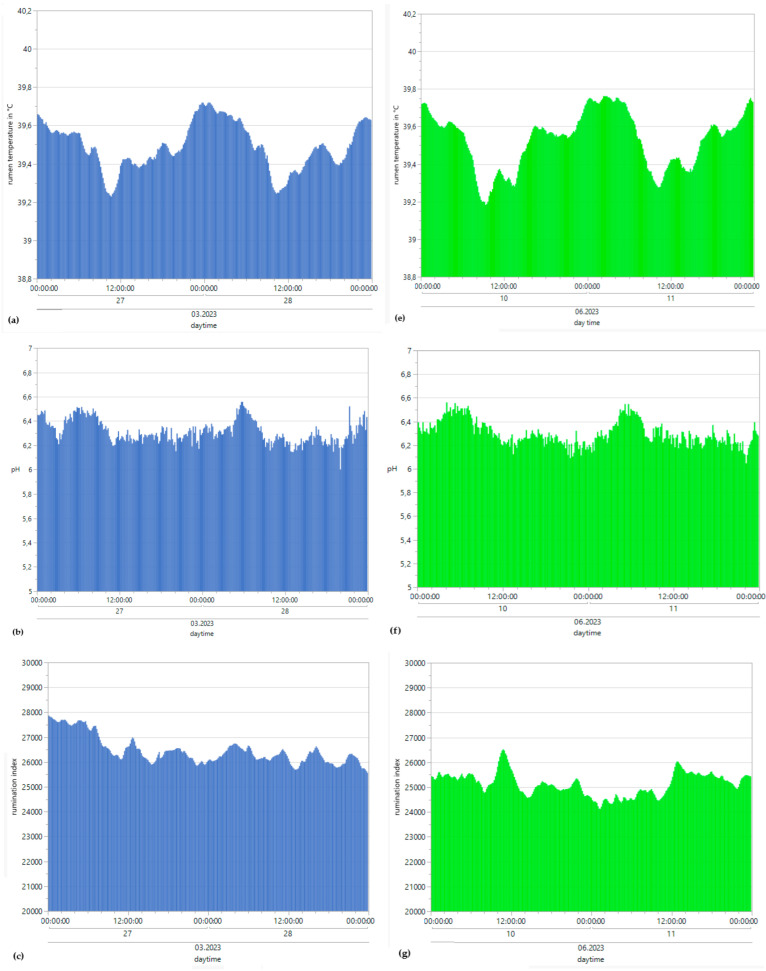

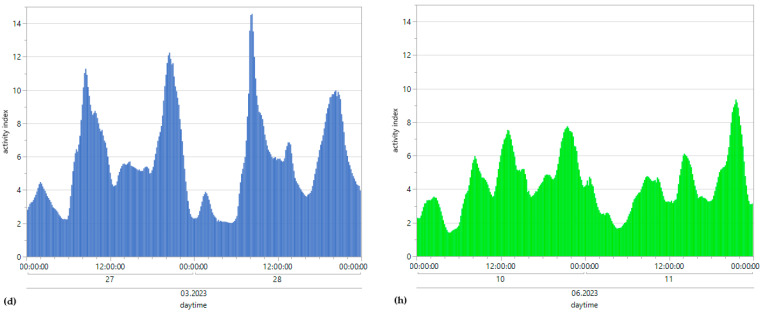
Means of reticulorumen temperature, pH, the rumination index and the activity index for all bulls under winter (**a**–**d**) and summer conditions (**e**–**h**).

**Figure 5:**
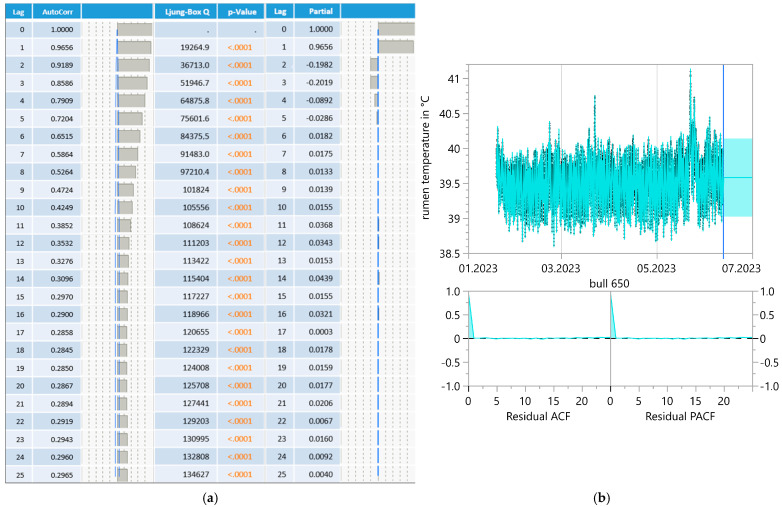
Correlogram (**a**) and Seasonal ARIMA (3, 0, 0)(11, 0, 0)2 forecast model (**b**) for bull reticulorumen temperature.

**Figure 6:**
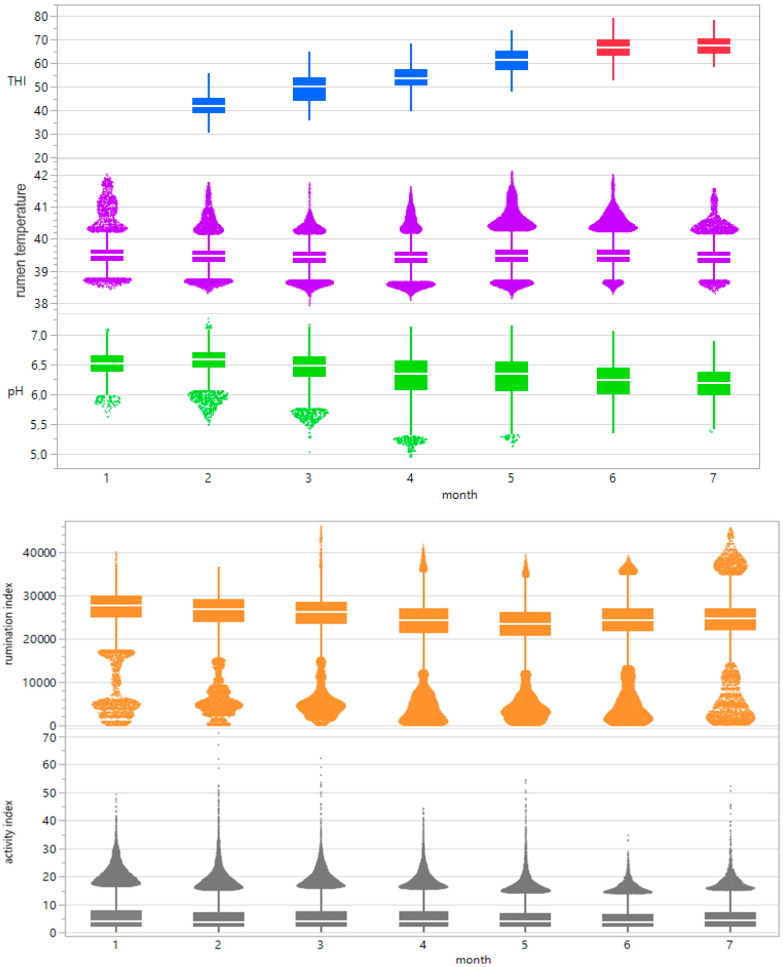
Boxplot progressions of the THI (temperature-humidity index), rumen temperature, rumen pH, rumination index and activity index across 7 months.

ReT significantly increased from 39.2 °C to 39.8 °C throughout the days under HS conditions (THI > 74) and for conditions without heat stress, with a maximum THI of approximately 53 (Figure 3). Notably, the rhythmic increase in ReT in all bulls clearly delayed the increase in THI. To determine the eventual relationships with the appearance of heat load, the average residuals of duration from the maximum THI to the maximum ReT were calculated, which would reveal possible increases in ReT on days with higher ambient temperatures (Table 1). The residuals alternated from 4 to 17 h under HS conditions to 5–15 h without HS. The highest values occurred between 10 pm and 2 pm, and the lowest occurred between 9 am and 1 pm. The development of ReT after THI maximum is depicted in Table 2. Two hours after THI maximum, positive and negative temperature differences are equally distributed. Four and six hours after THI maximum, a larger proportion of positive temperature differences occurs. That applies to comfort (THI ≤ 74) and alert conditions (74 < THI ≤ 78). The percentages of positive temperature differences after THI maximum are Td**_winter-ReT 2h_** = 46.93%, Td**_winter-ReT 4h_** = 59.18%, Td**_winter-ReT 6h_** = 71.43%, Td**_summer-ReT 2h_** = 42.86%, Td**_summer-ReT 4h_** = 57.14%, Td**_summer-ReT 6h_** = 75.51%. Table 3 and Table 4 depict the possible correlation between ReT range and daily THI_max_ values from individual bulls, differentiated according to summer and winter conditions, as determined by Pearson’s and Spearman’s correlations. The mean ReT differed only slightly between summer and winter conditions (Figure 4a,e). Daily fluctuations with minimum temperatures of approximately 39.2 °C and maximum temperatures of approximately 39.8 °C can be observed under both conditions. The collective mean ReT over the days assessed in winter (*n* = 14,971) was 39.48 °C, with a SD of 0.27 °C, a minimum mean of 38.31 °C and a maximum mean of 40.69 °C. In comparison, the collective mean ReT over the days assessed in summer (*n* = 14,030) was 39.53 °C, with a SD of 0.36 °C, a minimum mean of 38.39 °C and a maximum mean of 42.02 °C. Because we can map only the trend of the sensor bolus parameter over half a year, we decided to prepare a Seasonal ARIMA that allows us to predict for the complete year. This Seasonal ARIMA (3, 0, 0)(11, 0, 0)2 forecasted a constant graph level for ReT at 39.57 °C without variation between summer and winter (Figure 5, Table 5). The monthly mean values of ReT were steady throughout the winter and summer and did not significantly differ (Figure 6). Pearson’s correlations between the THI and ReT from all bulls throughout the entire experiment are described in Table 6.

### 3.2. Rumination

Minimum winter rumination occurred at 1964 s of rumination activity/24 h (RA/24 h), the maximum winter amplitude occurred at 46,090 RA/24 h, the minimum summer rumination occurred at 3129 RA/24 h, and the maximum summer rumination occurred at 36,524 R/24 h (Figure 4c,g). For collective mean rumination during winter conditions, the result was 26,453 RA/24 h, with a SD of 4752 RA/24 h. For summer conditions, the collective mean rumination was 25,132 R/24 h, with a SD of 4170 RA/24 h. The mean rumination index was higher during winter days but followed no specific rhythms or patterns. Table 3 and Table 4 depict the possible correlation between rumination index range and daily THI_max_ values from individual bulls, differentiated according to summer and winter conditions, as determined by Pearson’s and Spearman’s correlations.

The monthly mean rumination from all bulls tended to decrease slightly from February to May but increased beginning in June (Figure 6). Pearson’s correlations between the THI and rumination index from all bulls throughout the entire experiment are described in Table 6.

### 3.3. Rumen pH

The minimum winter pH was 5.4, and the maximum winter amplitude was 6.89. The minimum summer pH was 6.03, and the maximum summer pH was 6.56, with the highest values occurring during the early morning hours and the lowest occurring during the evening (Figure 4b,f). For the collective mean pH during winter conditions, the result was 6.31, with a SD of 0.24. For summer conditions, the collective mean pH was 6.3, with a SD of 0.3. Table 3 and Table 4 depict the possible correlation between rumen pH range and daily THI_max_ values from individual bulls, differentiated according to summer and winter conditions, as determined by Pearson’s and Spearman’s correlations. The monthly mean pH values for all bulls decreased from 6.5 to 6.2 from winter to summer (Figure 6). Pearson’s correlations between the THI and rumen pH from all bulls throughout the entire experiment are described in Table 6.

### 3.4. Motion Activity

The minimum winter motion activity index was 0.01, the maximum winter amplitude was 52.57, the minimum summer motion activity index was 0.01, and the maximum summer motion activity index was 27.78 (Figure 4d,h). For collective mean motion activity during winter conditions, the result was 5.8, with a SD of 4.6. For summer conditions, the collective mean motion activity was 4.3, with a SD of 3.5. The mean motion activity index was higher during the winter days but followed no specific rhythms or patterns. Table 3 and Table 4 depict the possible correlation between activity index range and daily THI_max_ values from individual bulls, differentiated according to summer and winter conditions, as determined by Pearson’s and Spearman’s correlations. The monthly mean values of our parameters were steady at 5.15 throughout the winter and summer and showed no significant differences or trends (Figure 6). Pearson’s correlations between the THI and activity index from all bulls throughout the entire experiment are described in Table 6.

## 4. Discussion

### 4.1. Reticulorumen Temperature

Heat load is characterized as “the thermal state where an animal must respond to environmental conditions physiologically (altered respiration, panting, and blood flow to the skin) or behaviorally (altered dry matter intake, activity, and lying time) to maintain thermal balance and normal functioning” [33]. Foroushani et al. (2022) reported that cattle maintain a steady body temperature (BT) as long as they can stay in a range of the “thermoneutral” zone [34]. Thermoregulatory mechanisms are effective only to a certain limit, beyond which hyperthermia occurs [35]. ReT is regarded as an effective measure of BT [36], which is the reason we chose it for our study. To interpret ReT correctly, we also have to focus on BT. The ability of thermoregulatory mechanisms to maintain BT within narrow limits has been verified [37,38]. The diurnal range of BT in healthy cattle in summer is greater than that in winter, suggesting that the circadian rhythm of BT changes because of the duration and magnitude of changes in heat/cold exposure and/or day length [39]. The mean daily BT can vary between and within individuals over time, which makes it difficult to define a range of normal BT in homeotherms. The BT of an individual animal may be within the ‘normal’ range for some combinations of species, age, physiological status and acclimatization, but the same temperature may indicate failure of homeothermy on other occasions. There are reports of a diphasic daily rhythm in cattle [40,41] and monophasic or polyphasic rhythms [41]. The diphasic rhythm is visible in our study, where ReT has its highest values between night from 10 pm to 2 pm and lowest values in the middle of the day between 9 am and 1 pm. Part of the variation between studies is probably due to differences in ultradian rhythms (more correctly called episodic ultradian events) between the cattle in the different studies [39,42]. The BT of each species of homeotherm varies within a defined range, and individuals within a species can differ. BT has a natural daily rhythm, oscillating by less than 1 °C each day [43,44,45]. Prendiville et al. [46] reported that compared with BT, ReT had a lower changeability during the day at other sites, such as rectal temperature or tympanic temperature. The results of our study support this hypothesis, with mean ReT oscillating by 0.5 °C each day, excluding drinking events.

The bolus system applied in this study was previously used to detect heat stress in German dairy cows and demonstrated that ReT “increased when the THI threshold of 65 was exceeded” [47]. However, it must be noted that, in particular, the interaction between high milk yields and ambient heat conditions led to an increase in ReT. Antanaitis et al. (2016) reported that ReT is influenced by circadian rhythm and season [48]. Furthermore, Woodward et al. (2024) developed a predictive model from the ReT of a rumen bolus and weather data using single and multiple linear regression in dairy cattle [49]. Within our study, the comparison of the monthly moving average of ReT = 39.57 °C with the actual daily mean of days with a THI > 74 did not provide sufficient evidence for a heat load indicator. We investigated the potential connection between the delay in the maximum amplitude of the THI and ReT. Given that the scattering of residual maximum hours is too high, we must assume that the daily maximum amplitude of ReT and its rhythm are more dependent on other factors (e.g., feeding cycles) than HL. This impression was enhanced by the increase in ReT four to six hours after the occurrence of the THI maximum values. This increase occurred under both conditions, with and without HL, which suggests that a circadian rhythm was also reflected in ReT. However, this does not provide proof of an influence of high ambient temperatures on rumen temperature. Although Liang et al. [50] reported that summer ReT was greater than spring, autumn, or winter ReT, our results could not verify this assertion. Daily ReT rhythms appeared equal under summer and winter conditions, and the expected increase in the mean ReT level under HL failed to appear. In dairy goats, rumen bolus sensor revealed that rumen temperature, rectal temperature, water intake and respiratory rate had a greater increase in heat stress than thermoneutral environments, which possibly indicates an altered microbial fermentation under high temperature conditions [51].

During the fattening period, it was only possible to observe a restricted period of time because the insertion of the bolus was not possible before the bulls gained a weight of 300 kg, which occurred at 10 months of age. Given that the fattening period is 17 months, this leads to a remaining trial period of 7 months. To display a full year, the Seasonal ARIMA was performed, and the results showed that the moving average of ReT = 39.57 °C was not influenced by the time of the year (*R*^2^ = 0.98). Considering that the moving average did not change throughout the year, we cannot confirm that ReT is an effective heat load indicator both in the early state and after HL occurred. Pearson’s correlation did not reveal a relationship between THI and ReT (*r* = −0.06; *p* < 0.001). For Pearson’s and Spearman’s correlation between ReT range and daily THI_max_ values from individual bulls, it should be noted, however, that even the largest significant correlations were mostly only within the weakly significant range of absolute < 0.5. This also suggests that there were no relationships present that would be suitable for an early detection of HL.

### 4.2. Rumination

Feeding behavior is subject to the influence of HL. Previous studies reported that calves had lower dry matter intake (DMI) during periods of HL [18,52]. Similar results were previously reported for Holstein heifers [53,54]. One possible explanation for the reduction in DMI could be that reduced gastrointestinal motility was accompanied by the presence of HS [55,56]. Rogers et al. also assumed that lower feeding behavior may be caused by the dilution of rumen contents caused by increased water intake in steers [57]. Several studies have shown that a decrease in the DMI reflects decreasing rumination times among dairy cows [22,58,59,60]. Idris et al. confirmed that this also applies to beef cattle [61]. The results of our experiment revealed that rumination, measured with a rumen sensor bolus, decreased with increasing ambient temperature in comparison to days with a THI < 74. A long-term effect was also visible when the monthly mean rumination from all bulls tended to decrease slightly from February to May and then increased beginning in June. Pearson correlation analysis revealed only a minimal correlation with the THI (*r* = −0.11; *p* < 0.001). Pearson’s and Spearman’s correlation between rumination index range and daily THI_max_ values from individual bulls revealed no significant correlation.

### 4.3. Rumen pH

Heat stress reduces the total production of volatile fatty acids with individual variation and results in changes in the ruminal pH [62]. Zhao et al. also reported a significant decrease in rumen pH in lactating Holstein dairy cows under HS [63]. Similar results were reported in other studies examining the rumen microbiome and pH [64,65]. Uyeno et al. profiled the changes in the rumen microflora composition of Holstein heifers during heat stress and reported consistent population-level changes in certain bacterial groups with heat stress, i.e., the relative populations of the Clostridium coccoides–Eubacterium rectale group and the genus Streptococcus increased, and that of the genus Fibrobacter decreased in response to increasing temperature [66]. Rumen acidosis can be caused by faster growth of lactate-producing bacteria such as Streptococcus bovis because lactate is absorbed much less by the rumen epithelium than volatile fatty acids such as acetate, propionate, and butyrate [67,68]. Our study confirmed this assumption for fattening bulls, as the mean pH values decreased throughout the summer months. Nevertheless, the comparison between daily fluctuations in pH values under HL failed to yield significant deviations from those captured on days of winter. Pearson correlation analysis revealed a weak negative linear relationship with the THI (*r* = −0.3; *p* < 0.001), which can be considered noteworthy. Pearson’s and Spearman’s correlation between rumen pH range and daily THI_max_ values from individual bulls revealed no significant correlation.

### 4.4. Motion Activity

Heinicke et al. (2019) reported that with increasing heat load, the lying time of dairy cows decreases, and the number of steps increases [69]. Further studies have confirmed that HL in dairy cows tends to increase their motion activity [22,70,71]. Idris et al. (2024) reported greater discomfort caused by HS for beef cattle, which implies longer standing and more stepping [61]. The data from the bolus system we used draws the opposite picture, showing that the activity index is lower on individual days with HL than on days without HL. The monthly means of the motion activity index over 7 months could also not verify that HL led to increasing activity because the means remained constant at a value of 5.15. Pearson correlation was not related to the THI (*r* = 0.04; *p* < 0.001). Pearson’s and Spearman’s correlation between activity index range and daily THI_max_ values from individual bulls revealed no significant correlation.

## 5. Conclusions

Conclusively, our study could not show that heat load has visible short-term effects on ReT or rumen pH, but rumination is reduced, and motion activity peaks are narrowed on days with high ambient temperatures. Apparently, ReT and pH are rather dependent on diurnal rhythms and, very likely, on feeding cycles. Our goal of using ReT as an early indicator to predict heat load could not be confirmed. Nevertheless, the results revealed a notable decrease in rumen pH and rumination with increasing high THI exposure over a longer period of time. Therefore, considering rumen sensor bolus data to investigate HL in retrospect represents a valid tool for farmers and researchers assessing animal welfare status.

## Figures and Tables

**Table 1:** Overview of times with a reticulorumen temperature maximum and its residuals to the THI (temperature-humidity index) maximum; timestamp Summer THI_max_ = 11:00 a.m.; timestamp Winter THI_max_ = 2:00 p.m.

Bull-Nmber	Timestamp Temp_max_ Summer Condition	Timestamp Temp_max_ Winter Condition	Hours THI_max_—Temp_max_ Summer Condition	Hours THI_max_—Temp_max_ Winter Condition
01	1:30 a.m.	3:10 a.m.	14:30	13:10
02	6:00 p.m.	7:00 p.m.	7:00	5:00
03	6:20 p.m.	1:50 a.m.	7:20	11:50
04	2:50 a.m.	12:40 a.m.	15:50	10:40
05	2:30 a.m.	12:00 a.m.	15:30	10:00
06	5:00 p.m.	12:00 a.m.	6:00	10:00
07	2:00 a.m.	11:50 p.m.	15:00	9:50
08	1:50 a.m.	10:20 p.m.	14:50	8:20
09	4:30 a.m.	1:40 a.m.	17:30	11:40
10	5:30 p.m.	3:30 a.m.	6:30	13:30
11	4:20 p.m.	3:40 a.m.	5:20	13:40
12	4:50 a.m.	4:00 a.m.	17:50	14:00
13	12:00 a.m.	11:30 p.m.	13:00	9:30
14	7:30 p.m.	4:00 a.m.	8:30	14:00
15	11:50 p.m.	1:20 a.m.	12:50	11:20
16	1:00 a.m.	10:10 p.m.	14:00	8:10
17	1:00 a.m.	10:30 p.m.	14:00	8:30
18	3:40 p.m.	1:40 a.m.	4:40	11:40
19	12:00 a.m.	12:50 a.m.	13:00	10:50
20	1:10 a.m.	12:20 a.m.	14:10	10:20
21	2:10 a.m.	1:00 a.m.	15:10	11:00
22	2:00 a.m.	12:50 a.m.	15:00	10:50
23	10:30 p.m.	9:50 p.m.	11:30	7:50
24	9:40 p.m.	11:30 p.m.	10:40	9:30
25	8:20 p.m.	2:30 a.m.	9:20	12:30
26	2:20 a.m.	0:40 a.m.	15:20	10:40
27	2:50 a.m.	9:40 p.m.	15.20	7:40
28	9:10 p.m.	3:10 a.m.	10:10	13:00
29	5:20 p.m.	2:10 a.m.	6:20	12:10
30	3:30 a.m.	1:20 a.m.	16:30	11:20
31	2:00 a.m.	4:40 a.m.	15:00	14:40
32	5:50 a.m.	1:50 a.m.	18:50	11:50
33	11:30 p.m.	12:10 a.m.	12.30	10:10
34	3:40 a.m.	3:00 a.m.	16:40	13:00
35	11:00 p.m.	7:20 p.m.	12:00	5:20
36	2:50 a.m.	12:30 a.m.	15:50	10:30
37	1:40 a.m.	11:10 p.m.	14:40	9:10
38	1:40 a.m.	2:40 a.m.	14:40	12:40
39	12:10 a.m.	3:00 a.m.	13:10	13:00
40	10:40 p.m.	2:10 a.m.	11:40	12:10
41	5:50 p.m.	11:00 p.m.	6.50	9:00
42	3:00 a.m.	3:00 a.m.	16:00	13:00
43	5:10 p.m.	4:20 a.m.	6:10	14.20
44	12:20 a.m.	2:20 a.m.	13:20	12:20
45	11:30 p.m.	3:00 a.m.	12:30	13:00
46	3:10 a.m.	3:20 a.m.	16:10	13:20
47	1:00 a.m.	2:20 a.m.	14:00	12:20
48	2:50 a.m.	2:30 a.m.	15:50	12:30
49	6:30 p.m.	12:10 a.m.	7:30	10:10

**Table 2:** Overview of reticulorumen temperature (ReT) differences during moments after THI maximum under winter and summer conditions; Term 0 h = ReT at the moment of THI maximum, Term 2 h = temperature difference to ReT two hours after THI (temperature-humidity index) maximum, Term 4 h = temperature difference to ReT four hours after THI maximum, Term 6 h = temperature difference to ReT six hours after THI maximum.

Winter					Summer			
Bull Number	Term 0 h in °C	Term 2 h in °C	Term 4 h in °C	Term 6 h in °C	Term 0 h in °C	Term 2 h in °C	Term 4 h in °C	Term 6 h in °C
01	38.97	+0.52	+0.25	+0.35	39.35	−0.29	+0.04	−0.02
02	39.73	−0.4	−0.64	+0.35	39.06	+0.1	+0.35	+0.48
03	39.58	−0.01	+0.13	+0.03	39.55	−0.06	+0.35	+0.4
04	39.26	−0.13	−0.45	−0.15	39.17	±0	+0.21	−0.04
05	39.18	+0.55	+0.39	+0.26	39.28	−0.12	−0.22	+0.39
06	39.31	−0.18	+0.06	±0	39.25	+0.28	+0.2	+0.2
07	39.4	+0.16	−0.17	+0.06	39.1	+0.03	+0.2	+0.35
08	39.39	±0	−0.18	+0.29	39.41	+0.11	+0.19	+0.35
09	39.07	+0.44	+0.03	+0.33	39.47	−0.19	+0.32	+0.44
10	39.44	−0.05	+0.14	+0.3	38.95	−0.08	+0.27	+0.74
11	39.72	−0.41	−0.37	−0.4	39.18	+0.09	−0.01	+0.46
12	39.53	−0.08	−0.04	+0.02	39.24	−0.18	+0.19	+0.26
13	39.36	−0.25	+0.07	−0.19	38.85	+0.22	+0.31	+0.65
14	39.42	−0.07	−0.13	+0.37	39.69	−0.57	−0.11	±0
15	39.95	−0.21	−0.16	−0.72	39.52	−0.08	+0.24	−0.09
16	39.23	+0.16	+0.29	+0.68	39.73	−0.05	−0.1	−0.17
17	39.53	+0.1	+0.22	+0.13	39.84	−0.16	−0.8	+0.19
18	39.03	+0.42	+0.17	+0.34	39.33	−0.03	−0.1	+0.06
19	39.56	−0.16	+0.13	+0.12	39.22	−0.12	−0.03	+0.27
20	39.66	−0.05	−0.36	−0.32	39.13	+0.53	+0.39	+0.68
21	39.25	+0.21	+0.11	+0.19	39.36	−0.21	−0.15	−0.06
22	39.17	+0.22	+0.21	+0.31	39.49	−0.18	−0.44	−0.13
23	39.14	+0.29	+0.32	+0.59	39.63	−0.06	−0.11	−0.15
24	39.52	+0.12	+0.21	+0.02	39.45	+0.01	−0.13	+0.08
25	39.26	+0.22	−0.03	+0.66	39.11	+0.42	+0.3	+0.89
26	39.47	−0.32	+0.09	±0	39.08	+0.15	−0.01	+0.23
27	39.8	−0.35	±0	−0.15	39.34	+0.04	+0.32	+0.05
28	39.85	−0.18	+0.11	−0.11	39.54	+0.12	−0.19	−0.1
29	39.39	−0.04	−0.09	+0.06	39.51	+0.42	+0.86	+1.07
30	39.55	−0.43	+0.09	−0.32	38.77	+0.58	+0.44	+0.39
31	39.52	+0.12	−0.14	+0.04	39.15	+0.44	+0.24	+0.43
32	39.81	−0.24	−0.24	+0.15	39.42	+0.23	+0.21	−0.07
33	39.53	+0.14	+0.06	+0.13	39.15	+0.15	+0.17	+0.34
34	39.09	+0.51	+0.44	+0.51	39.23	−0.25	+0.04	+0.41
35	39.81	−0.27	+0.34	−0.16	39.49	−0.32	−0.03	+0.24
36	39.3	+0.43	+0.24	+0.13	39.66	−0.38	−0.1	+0.14
37	39.58	−0.33	−0.11	+0.14	39.68	−0.44	±0	−0.47
38	39.22	−0.3	+0.2	+0.12	39.67	−0.53	+0.05	−0.03
39	39.01	+0.49	−0.03	+0.27	39.24	+0.02	−0.01	+0.21
40	39.32	+0.27	+0.11	+0.04	39.68	−0.31	+0.03	+0.01
41	39.6	−0.16	−0.2	−0.25	39.57	−0.28	−0.04	+0.42
42	39.23	+0.46	+0.35	−0.06	39.48	−0.49	−0.13	+0.14
43	39.09	+0.65	+0.82	+0.22	39.53	−0.3	−0.02	+0.12
44	39.35	+0.16	−0.16	+0.07	39.23	−0.28	−0.06	+0.02
45	40.07	−0.39	+0.23	+0.39	39.02	+0.24	+0.24	+0.51
46	39.43	−0.27	−0.21	−0.17	39.06	+0.02	+0.11	+0.2
47	39.6	−0.12	−0.27	+0.04	39.4	−0.16	+0.29	+0.19
48	39.05	+0.37	+0.06	+0.18	39.24	−0.02	+0.18	+0.34
49	39.12	+0.36	+0.42	+0.02	39.73	+0.34	+0.94	+0.84

**Table 3:** Pearson’s correlation between rumen sensor bolus parameter ranges and daily THI**_max_** values from individual bulls differentiated according to summer and winter conditions; ssn = Season (W = winter, corresponds to measurements up from January to April; S = summer, corresponds to all measurements from May to July), min d = minimum number of daily values considered for a bull (per season), max d = maximum number of daily values considered for a bull (per season), N sig 5pct = number of bulls for which a significant correlation (*p* < 0.05) was found (per season), min PCorr = minimum (smallest) value of significant correlations, med PCorr = median value of significant correlations, max PCorr = maximum (largest) value of significant correlations.

Trait 1	Trait 2	ssn	n Bulls	Min d	Max d	N sig 5pct	Min PCorr	Med PCorr	Max PCorr
activity index range	max daily THI	S	59	15	71	9	−0.43203	0.26824	0.40298
activity index range	max daily THI	W	60	22	77	9	−0.34453	−0.30118	0.48130
ReT range	max daily THI	S	59	15	71	5	−0.40945	−0.26388	0.36395
ReT range	max daily THI	W	60	22	77	7	−0.22909	0.37505	0.44096
rumen pH range	max daily THI	S	9	39	71	4	−0.41504	−0.29436	0.27815
rumen pH range	max daily THI	W	10	22	77	1	−0.43084	−0.43084	−0.43084
rum. index range	max daily THI	S	55	6	71	2	0.30339	0.48541	0.66744
rum. index range	max daily THI	W	60	15	77	0			

ReT = reticulorumen temperature, rum. = rumination, THI = temperature-humidity index.

**Table 4:** Spearman’s correlation between rumen sensor bolus parameter ranges and daily THI_max_ values from individual bulls differentiated according to summer and winter conditions; min SCorr = minimum (smallest) value of significant (Spearman rank) correlations, med SCorr = median value of significant (Spearman rank) correlations, max SCorr = maximum (largest) value of significant (Spearman rank) correlations.

Trait 1	Trait 2	ssn	n Bulls	Min d	Max d	N sig 5pct	Min SCorr	Med SCorr	Max SCorr
activity index range	max daily THI	S	59	15	71	11	−0.48610	0.24700	0.46900
activity index range	max daily THI	W	60	22	77	12	−0.37996	−0.28554	0.43511
ReT range	max daily THI	S	59	15	71	6	−0.40462	−0.27944	0.34567
ReT range	max daily THI	W	60	22	77	7	0.26558	0.39242	0.46802
rumen pH range	max daily THI	S	9	39	71	5	−0.39465	−0.27694	0.32057
rumen pH range	max daily THI	W	10	22	77	1	−0.44188	−0.44188	−0.44188
rum. index range	max daily THI	S	55	6	71	1	−0.32109	−0.32109	−0.32109
rum. index range	max daily THI	W	60	15	77	2	0.39406	0.39974	0.40543

ReT = reticulorumen temperature, rum. = rumination, ssn = season, THI = temperature-humidity index.

**Table 5:** Model summary of Seasonal ARIMA (3, 0, 0)(11, 0, 0)2 for bull reticulorumen temperature.

Measure	Value
Degrees of Freedom	20,646
Sum of Squared Innovations	30.7863478
Sum of Squared Residuals	30.7873297
Variance Estimate	0.00149115
Standard Deviation	0.03861545
Akaike’s ‘A’ Information Criterion	−75,812.976
Schwarz’s Bayesian Criterion	−75,693.936
R Square	0.98016153
R Square Adj.	0.98014808
MAPE	0.07069371
MAE	0.02794955
–2LogLikelihood	−75,842.975

MAPE = Mean Absolute Percentage Error, MAE = Mean Absolute Error.

**Table 6:** Pearson’s correlations between the THI (temperature-humidity index) and the rumen sensor bolus parameters (ReT = reticulorumen temperature) from all bulls throughout the entire experiment.

Sensor Parameter	n	Mean	sd	Corr.–Coefficient THI (r)	*p* Value
ReT	941,078	39.46	0.32	−0.06	<0.001
rumen pH	131,188	6.33	0.32	−0.3	<0.001
rumination index	785,059	24,300.23	5726.85	−0.11	<0.001
motion activity	937,768	5.15	4.25	0.04	<0.001

## Data Availability

None of the data have been deposited in an official repository. Information can be provided by the authors upon request.
